# 1p36 deletion syndrome: first case report in Morocco detected by fluorescence *in situ* hybridization

**DOI:** 10.11604/pamj.2020.37.349.26166

**Published:** 2020-12-16

**Authors:** Kenza Dafir, Fatima Zahra Bouzid, Maria Mansouri, Nisrine Aboussair

**Affiliations:** 1 Genetics Department, Clinical Research Center, University Hospital Center Mohammed VI, Marrakesh, Morocco,; 2 School of Medicine and Pharmacy of Marrakech, Cadi Ayyad University, Marrakesh, Morocco

**Keywords:** 1p36 deletion, fluorescence in situ hybridization, facial dysmorphia, delayed development, case report

## Abstract

The 1p36 deletion syndrome results from a heterozygous deletion of the terminal chromosomal band of the short arm of chromosome 1. Monosomy 1p36 is the most common terminal deletion observed in men (1 in 5000 newborns), characterized by distinctive dysmorphia, delayed growth, psychomotor retardation, intellectual deficit, epilepsy and heart defects. Fluorescence in situ hybridization (FISH) and comparative genomic hybridization (CGH-array) are currently the two best diagnostic techniques. The objective of this work is to take stock of the first Moroccan case of 1p36 deletion and to illustrate the role of the geneticist in the diagnosis and management of this syndrome. There is currently no effective medical treatment for this disease.

## Introduction

1p36 deletion syndrome (OMIM: 607872) is a chromosomal abnormality characterized by intrauterine growth retardation, a characteristic facial dysmorphism made up of straight eyebrows, sunken eyes, wide and flat nasal bridge, upper floor middle of the hypoplastic face, a long philtrum, a pointed chin and a frequent delay in closing the anterior fontanel (>3cm at birth), microbrachycephaly, upturned ears of low implantation and malformed, brachydactyly, camptodactyly, short feet, hypotonia, developmental delay, intellectual deficit, seizures, heart defects and hearing and vision impairment. It is considered to be the most common terminal deletion in humans [1,2]. It is estimated that the syndrome occurs in one in every 5,000 to 10,000 births without differentiation of sex or ethnicity [1,3]. The syndrome is caused by a partial heterozygous deletion of the most distal band of the short arm of chromosome 1 (1p36), with breakpoints ranging from 1p36.13 to 1p36.33. Fifty percent of the cases are de novo terminal deletion, 29% are the interstitial deletion and the other cases are secondary to more complex chromosomal rearrangements.

We report the first Moroccan observation of 1p36 deletion referred to the medical genetic department of the Mohammed VI University Hospital of Marrakech for a dysmorphic syndrome, seizures, delayed growth, hypothyroidism and hypotonia. The objective of this case reports is to focus on the first Moroccan case of 1p36 deletion and to illustrate the role of the geneticist in the diagnosis, management and development of genetic counseling for this syndrome.

## Patient and observation

**Figure 1:**
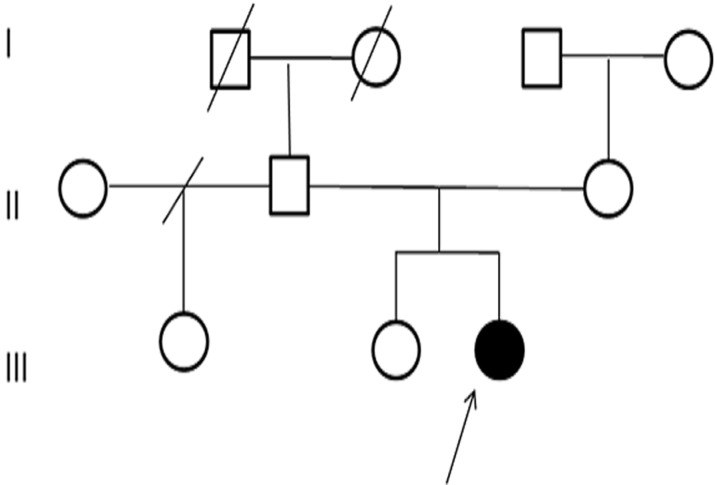
family pedigree

Female patient, from Morocco, has 3 years, non-consanguineous, the second of two siblings (Figure 1), was referred to the medical genetic department of the Mohammed VI University Hospital in Marrakech for a dysmorphic syndrome, seizures, delayed growth, hypothyroidism and hypotonia. The parents and the older sister were healthy, 38-year-old mother and 40-year-old father. The girl was resulting from a followed pregnancy, carried to term, without notion of teratogenic drug intake. The delivery took place by cesarean section (oligo-amnios + acute fetal pain), with intra-uterine growth retardation: birth weight=1 Kg 850, size=44 cm, cranial perimeter=33 cm and neonatal hypotonia. The girl presented a neonatal infection, developmental delay, transient hypothyroidism treated for 05 months with the notion of a single convulsive attack at the age of 05 months without treatment.

**Figure 2:**
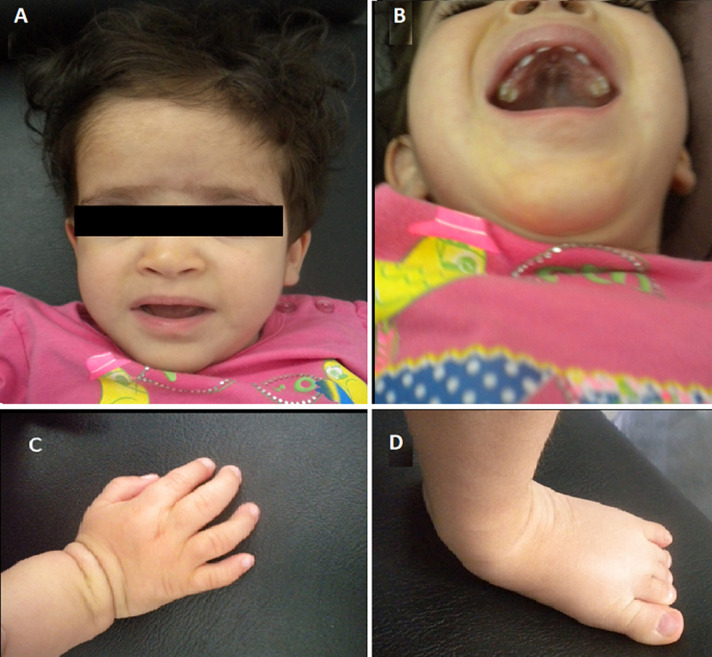
clinical characteristics of our patient: A) facial phenotype; B) ogival palate; C) brachydactyly and clinodactyly of the fifth finger; D) valgus

The clinical examination revealed: a microcephaly (cranial perimeter=44 cm (-2 standard deviation)), low body weight (weight=7 Kg (-3 standard deviation)),short stature (size=80 cm (-2 standard deviation)); hypotonia, the horizontal eyebrows, sunken eyes, no space between the eyebrows and the eyes, a worried look, a hypertelorism, the broad root of the nose, midface hypoplasia, the erased philtrum an ogival palate, brachydactyly, clinodactyly of the fifth finger and a bilateral valgus of the feet. The patient underwent whole skeleton radiographs showing delayed bone age. There were no other malformations or organ dysfunctions (Figure 2).

**Figure 3:**
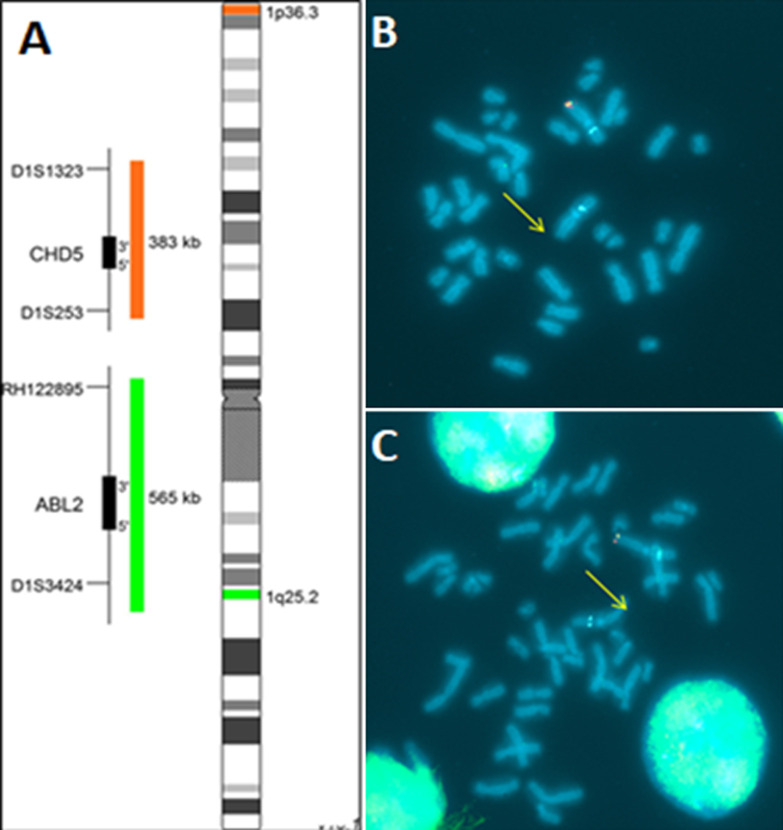
(A,B,C) study of fluorescence in situ hybridization (FISH) in the metaphase showing terminal microdeletion of chromosome 1, short arm (p), lane 6, region 3 (1p36) (arrows)

In front of the facial dysmorphia and other clinical features as well as the radiological explorations the diagnosis to evoke in our patient is the deletion syndrome 1p36. A molecular cytogenetic study, by fluorescence in situ hybridization (FISH), specific locus probe set (LSI p58 (1p36) spectrum orange/telvysion 1p spectrum green/LSI 1q25 spectrum aqua) was performed and confirmed the presence of a heterozygous microdeletion in 1p36: ish del (1) (p36p36) (p58-/tel 1p-) (Figure 3).

## Discussion

The 1p36 deletion syndrome is the most common deletion syndrome, the first case was described in 1981 [4]. The phenotype of the syndrome was defined on the basis of the most common characteristics: a recognizable cranio-facial dysmorphia (straight eyebrows, sunken eyes, a broad and flat nasal bridge, a middle part of the hypoplastic face, a long philtrum, a pointed chin and a frequent delay in closing the anterior fontanel, microbrachycephaly and upturned ears with low and malformed implantation), brachydactyly, camptodactyly, short feet, congenital hypotonia leading to difficulty swallowing, delayed development motor and motor skills and an language delay. A variable degree of intellectual deficit is observed in all patients. There are other manifestations like intrauterine growth retardation, congenital heart disease, hearing loss, ophthalmological, skeletal, external genital anomalies and more rarely kidney and thyroid abnormalities [5].

In 2007, a group of author concluded that there is no correlation between the size of the deletion and clinical signs in the 134 subjects reviewed [6]. They explained this by the fact that most of the genes associated with the 1p36 monosomy phenotype are at the distal end of the chromosome. This syndrome is due to a partial heterozygous deletion on the distal part of the short arm of chromosome 1, not detected by conventional cytogenetics. However, comparative genomic hybridization and analysis by fluorescence in situ hybridization (FISH) confirmed this diagnosis with breakpoints ranging from 1p36.13 to 1p36.33. A majority of cases (52 to 67%) have a terminal deletion of chromosome 1. Interstitial deletions (9.7-29%) and other abnormalities, such as derived chromosomes (7-16.4%) and complex rearrangements are less common [7,8].

The 1p36 genes most strongly involved in the 1p36 deletion syndrome essentially include: MMP23B (OMIM: 603321, large, late-closing anterior fontanel), GABRD (OMIM: 137163, neurodevelopmental abnormalities, neuropsychiatric problems, seizures), SKI (OMIM: 64780, developmental delay, intellectual disability, seizures, orofacial clefting, congenital heart defects), PRDM16 (OMIM: 605557, left vertricular non compaction, dilated cardiomyopathy), KCNAB2 (OMIM: 601142, developmental delay, intellectual disability, seizures), RERE (OMIM: 605226, short stature, developmental delay, intellectual disability, brain anomalies, vision problems, hearing loss, renal anomalies, congenital heart defects, cardiomyopathy), UBE4B (OMIM: 613565, cardiomyopathy and neurodevelopmental phenotypes), CASZ1 (OMIM: 609895, congenital heart defects and cardiomyopathy), PDPN ( OMIM: 608863, congenital heart defects, cardiomyopathy), SPEN (OMIM: 613484, congenital heart defects, cardiomyopathy, short stature, neurodevelopmental phenotypes), ECE1 (OMIM: 600423, congenital heart defects), HSPG2 (OMIM: 142461, cleft palate, congenital heart defects) and LUZP1 (OMIM: 601422, congenital heart defects, cleft palate, brain anomalies). Most of the genes have been identified by a combination of molecular cytogenetic mapping and the sequencing of candidate genes in humans or animals [9].

It is noteworthy that the deletion of other genes such as CDC2L2, TNFRSF25, AJAP1, TP73, CAMTA1, can contribute to the development of malignant tumors [10]. 1p36 syndrome must be differentiated from Rett, Agelman, Prader-willi and Aicardi syndrome, because some symptoms of the 1p36 deletion overlap with those of these syndromes [11]. If there is a family history, prenatal testing is possible by amniocentesis or chorionic villus sampling and cytogenetic analysis. Preimplantation genetic diagnosis is available for couples in which one parent is a known carrier and in this case the genetic counseling is recommended to inform parents of the risk of recurrence. Currently, there is no effective treatment other than symptomatic management, in particular neuropsychological follow-up.

## Conclusion

The identification of this micro-deletion allowed us to confirm the diagnosis in this patient, guide her clinical management and formulate adequate genetic counseling for the parents. Researchers' efforts still continue to provide important evidence for the contribution of the different genes involved in this syndrome and to identify a complete deletion/phenotype map for the 1p36 region that will allow physicians to provide the prognostic information desired by families and generate individualized care plans for patients with these deletions.
